# Proteomics and relationship with axonal pathology in multiple sclerosis: 5-year diffusion tensor imaging study

**DOI:** 10.1093/braincomms/fcad183

**Published:** 2023-06-13

**Authors:** Dejan Jakimovski, Ferhan Qureshi, Murali Ramanathan, Victor Gehman, Anisha Keshavan, Kelly Leyden, Michael G Dwyer, Niels Bergsland, Bianca Weinstock-Guttman, Robert Zivadinov

**Affiliations:** Buffalo Neuroimaging Analysis Center, Department of Neurology, Jacobs School of Medicine and Biomedical Sciences, University at Buffalo, State University of New York, Buffalo, NY 14203, USA; Octave Bioscience, Menlo Park, CA 94025, USA; Department of Pharmaceutical Sciences, University at Buffalo, State University of New York, Buffalo, NY 14214, USA; Octave Bioscience, Menlo Park, CA 94025, USA; Octave Bioscience, Menlo Park, CA 94025, USA; Octave Bioscience, Menlo Park, CA 94025, USA; Buffalo Neuroimaging Analysis Center, Department of Neurology, Jacobs School of Medicine and Biomedical Sciences, University at Buffalo, State University of New York, Buffalo, NY 14203, USA; Buffalo Neuroimaging Analysis Center, Department of Neurology, Jacobs School of Medicine and Biomedical Sciences, University at Buffalo, State University of New York, Buffalo, NY 14203, USA; IRCCS, Fondazione Don Carlo Gnocchi, Milan 20113, Italy; Jacobs Comprehensive MS Treatment and Research Center, Department of Neurology, Jacobs School of Medicine and Biomedical Sciences, University at Buffalo, State University of New York, Buffalo, NY 14203, USA; Buffalo Neuroimaging Analysis Center, Department of Neurology, Jacobs School of Medicine and Biomedical Sciences, University at Buffalo, State University of New York, Buffalo, NY 14203, USA; Center for Biomedical Imaging at the Clinical Translational Science Institute, University at Buffalo, State University of New York, Buffalo, NY 14203, USA

**Keywords:** proteomics, multiple sclerosis, axonal injury, diffusion tensor imaging

## Abstract

Blood-based biomarkers can be economic and easily accessible tools for monitoring and predicting disease activity in multiple sclerosis. The objective of this study was to determine the predictive value of a multivariate proteomic assay for concurrent and future microstructural/axonal brain pathology in a longitudinal study of a heterogeneous group of people with multiple sclerosis. A proteomic analysis was obtained on serum samples from 202 people with multiple sclerosis (148 relapsing-remitting and 54 progressive) at baseline and 5-year follow-up. The concentration of 21 proteins related to multiple pathways of multiple sclerosis pathophysiology was derived using Proximity Extension Assay on the Olink platform. Patients were imaged on the same 3T MRI scanner at both timepoints. Тhe rate of whole brain, white matter and grey matter atrophy over the 5-year follow-up was determined using the multi-timepoint Structural Image Evaluation, using Normalisation, of Atrophy algorithms. Lesion burden measures were also assessed. The severity of microstructural axonal brain pathology was quantified using diffusion tensor imaging. Fractional anisotropy and mean diffusivity of normal-appearing brain tissue, normal-appearing white matter, grey matter, T2 and T1 lesions were calculated. Age, sex and body mass index-adjusted step-wise regression models were used. Glial fibrillary acidic protein was the most common and highest-ranked proteomic biomarker associated with greater concurrent microstructural central nervous system alterations (*P* < 0.001). The rate of whole brain atrophy was associated with baseline levels of glial fibrillary acidic protein, protogenin precursor, neurofilament light chain and myelin oligodendrocyte (*P* < 0.009), whereas grey matter atrophy was associated with higher baseline neurofilament light chain, higher osteopontin and lower protogenin precursor levels (*P* < 0.016). Higher baseline glial fibrillary acidic protein level was a significant predictor of future severity of the microstructural CNS alterations as measured by normal-appearing brain tissue fractional anisotropy and mean diffusivity (standardized β = −0.397/0.327, *P* < 0.001), normal-appearing white matter fractional anisotropy (standardized β = −0.466, *P* < 0.0012), grey matter mean diffusivity (standardized β = 0.346, *P* < 0.011) and T2 lesion mean diffusivity (standardized β = 0.416, *P* < 0.001) at the 5-year follow-up. Serum levels of myelin-oligodendrocyte glycoprotein, neurofilament light chain, contactin-2 and osteopontin proteins were additionally and independently associated with worse concomitant and future axonal pathology. Higher glial fibrillary acidic protein levels were associated with future disability progression (Exp(B) = 8.65, *P* = 0.004). Multiple proteomic biomarkers are independently associated with greater severity of axonal brain pathology as measured by diffusion tensor imaging in multiple sclerosis. Baseline serum glial fibrillary acidic protein levels can predict future disability progression.

## Teaser

Jakimovski *et al*. utilized serum proteomic data consisting of 21 biomarkers related to multiple sclerosis pathophysiological processes in predicting concurrent and future microstructural MRI pathology. Higher glial fibrillary acidic protein and lower myelin-oligodendrocyte levels were predictive of more severe axonal damage.

## Graphical Abstract

**Graphical Abstract:**
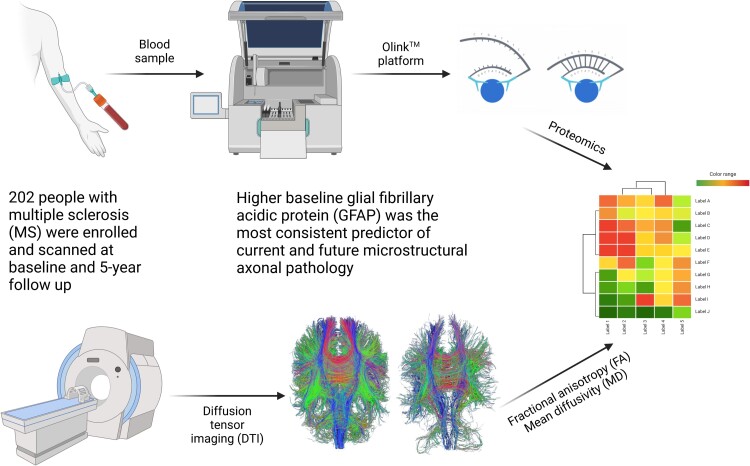

## Introduction

Multiple sclerosis is a neuroinflammatory and neurodegenerative disease of the CNS that commonly leads to physical and cognitive disability. The hallmarks of multiple sclerosis pathophysiology include antigen sensitization causing peripheral immune activation, and infiltration of lymphocytes through the blood–brain barrier (BBB) that ultimately lead to the destruction of the myelin sheets and axonal transection.^1^ Axonal transection propagates retrograde degeneration and results in considerable neuronal loss.^1^ Brain pathology in people with multiple sclerosis showed up to 39% fewer neurons when compared to healthy controls, while up to 40% of axons were demyelinated.^2^ These changes can be visualized through magnetic resonance imaging (MRI) as greater brain tissue atrophy and the presence of hyperintense T2 lesions, respectively.^2,3^ Such imaging biomarkers are routinely used as paraclinical outcomes in clinical trials and guide routine patient care.^4,5^

In addition to conventional and non-conventional MRI outcomes, there is an increased interest in the use of blood-based biomarkers.^6^ In comparison to long and expensive imaging sessions, these biomarkers can be conveniently obtained through frequent blood draws. Therefore, the development of a comprehensive, proteomic assay panel with reliable prognostic and treatment-response assessment may significantly improve the extent to which people with multiple sclerosis are routinely monitored. Serum neurofilament light chain (NfL) is one such promising biomarker, which reflects the amount of axonal pathology and acute inflammation.^7^ In particular, NfL levels are highly sensitive and increase at times of clinical relapse and radiologic activity when compared to people with multiple sclerosis in remission.^8^ However, NfL is non-specific to multiple sclerosis pathology, and its values can be influenced by axonal injury of any aetiology.^9^ The increase of NfL levels with normal aging further diminishes its stand-alone utility as an multiple sclerosis-specific biomarker.^9^ The ability to concurrently measure several biomarkers that represent multiple sclerosis-specific pathophysiologic pathways using a multivariate proteomic panel could circumvent the limitations of NfL. While the existing multiple sclerosis literature has primarily focused on the predictive value of individual serum proteins, comprehensive multivariate proteomic studies are less common. In addition, previous studies explored the relationship between proteomic biomarkers and conventional inflammatory and neurodegeneration MRI measures (lesions and brain atrophy), which lack specificity for determining the pathological substrate of underlying damage, and in particular, those indices reflecting axonal loss.^10,11^

Diffusion tensor imaging (DTI) is a non-conventional MRI measure that describes the diffusion of water molecules.^12^ Since axons and their myelin sheet mainly constrain the water to one axis, the brain is characterized by anisotropic diffusion properties.^12^ The destruction of physical barriers allows the water to more easily diffuse in multiple directions and the transition from anisotropic to isotropic diffusion can be quantified using DTI-derived measures such as fractional anisotropy (FA) and mean diffusivity (MD).^12^ Histopathological and DTI studies have previously corroborated the underlying substrate of DTI abnormalities as possible axonal loss in the lesioned tissue and microglial activation in the normal-appearing white matter (NAWM).^13,14^ Therefore, we specifically applied DTI in this study as it is one of the most well-established MRI outcomes of axonal loss in multiple sclerosis.

Based on this background, we aimed at determining the utility of a multivariate proteomic assay in predicting concurrent and future axonal pathology in a heterogeneous group of people with multiple sclerosis. We hypothesized that the cerebral microstructural integrity in multiple sclerosis, as measured by DTI, can be better explained by multiple proteomic biomarkers that stem from different pathophysiological pathways related to myelin biology, neuroaxonal integrity, immunomodulation and neuroinflammation.

## Materials and methods

### Study population

The study population utilized in this analysis was part of a previously completed longitudinal study that explored the cardiovascular, environmental and genetic factors in multiple sclerosis (CEG-MS).^10^ For this particular analysis, the inclusion criteria were as follows: (i) age of 18–75 years old at baseline timepoint; (ii) diagnosed with multiple sclerosis or clinically isolated syndrome (CIS), per 2010-revision of the McDonald criteria;^15^ (iii) availability of baseline serum sample, MRI and clinical assessment within 30 days of each other; and (iv) availability of an MRI and clinical examination at ∼5 years (±6 months) follow-up. An optional serum sample at the 5-year follow-up was utilized if available. The exclusion criteria for the people with multiple sclerosis were as follows: (i) having a clinical relapse or administered intravenous corticosteroid therapy in the 30 days preceding MRI and blood draw; and (ii) being unable to undergo any of the aforementioned study procedures. The people with multiple sclerosis were examined by a board-certified neurologist, and their disability was scored using the Expanded Disability Status Scale (EDSS) score.^16^ A structured questionnaire was used to collect demographic and clinical information. Details regarding the disease-modifying therapy (DMT) at baseline and follow-up visits were collected and stratified based on the mechanism of action and/or the European Medicines Agency potency criteria. Out of the DMTs used, only natalizumab was considered a high-potency DMT, whereas the remaining DMTs (interferon-β, glatiramer acetate, fingolimod, teriflunomide, dimethyl fumarate and off-label therapies) were considered as moderate potency. Based on the clinical presentation and disease history, the people with multiple sclerosis were categorized into relapsing-remitting multiple sclerosis and progressive multiple sclerosis.^17^ Due to the low sample size of primary-progressive multiple sclerosis cases, both secondary and primary-progressive multiple sclerosis were merged into one group. Disability progression (DP) in the people with multiple sclerosis was determined using standard clinical trial criteria^18^ where (i) EDSS change of ≥1.0 point if the baseline EDSS was between 1.0 and 5.0; and 2) EDSS change of ≥0.5 points if the baseline EDSS was ≥5.5. People with multiple sclerosis with a baseline EDSS of 0 had to reach an EDSS of 2.0 in order to be considered a disability progressor. People with multiple sclerosis not fitting the DP criteria were labelled as stable. The CEG-MS study and the retrospective proteomic analyses were approved by the University Institutional Review Board, and all subjects provided written informed consent.

### Proteomics analyses

All blood (serum) samples were processed within 24 hours from acquisition and stored at −80°C until analyses. No freeze and thaw cycles were performed in the interim period. All samples were sent to Octave Bioscience (Menlo Park, CA, USA) for proteomic analysis using a custom-developed multiple sclerosis disease activity (MSDA) assay panel. Proteomic analysis was performed blinded to the demographic, clinical and MRI data. The MSDA assay uses Proximity Extension Assay methodology and is performed on the Olink™ platform. Twenty-one (21) proteins that are associated with key biological pathways of multiple sclerosis pathophysiology were selected for inclusion on the panel based on results from discovery analyses investigating the relative expression of 1196 proteins in previously characterized multiple sclerosis cohorts. The MSDA score utilizes a stacked classifier logistic regression model of 18 age- and sex-adjusted protein concentrations as previously described to determine four disease pathway scores (immunomodulation, neuroinflammation, myelin biology and neuroaxonal integrity) as well as an overall disease activity score.^19^ The complete list of proteins (with commonly used aliases and their abbreviations) is shown in Table 1. The MSDA scores were validated for the prediction of inflammatory activity such as relapses and gadolinium-enhancing lesions. None of the samples were diluted. The assay was optimized and analytically validated with undiluted serum samples as the intended sample matrix.^19^ Both parallelism and inter-assay coefficients of variability (CV) have been recently reported in the literature.^19^

**Table 1:** List of proteomic biomarkers analyses using the multivariate assay

Marker	Name (Alias)
NfL	Neurofilament light
MOG	Myelin-oligodendrocyte glycoprotein
CD6	Cluster of Differentiation 6
CXCL13	C-X-C Motif Chemokine Ligand 13, BLC
CXCL9	Monokine induced by gamma interferon, MIG
CDCP1	CUB domain-containing protein 1
CCL20	MIP-3 alpha
OPG	Osteoprotegerin, TNFRSF11B
IL-12B	Interleukin 12B
APLP1	Amyloid beta precursor like protein 1
GH	Somatotropin, growth hormone
VCAN	Versican, versican proteoglycan
TNFRSF10A	TRAILR1, DR5—Death Receptor 5
COL4A1	Collagen alpha-1 (IV) chain
SERPINA9	Serpin family A member 9
PRTG	Protogenin
FLRT2	Leucine-rich repeat transmembrane protein
TNFSF13B	BAFF
OPN	Osteopontin
CNTN2	Contactin 2
GFAP	Glial fibrillary acidic protein

BLC, B lymphocyte chemoattractant; MIG, monokine induced by gamma interferon; MIP-3, macrophage inflammatory protein-3; BAFF, B-cell activating factor.

In particular, mixing two undiluted serum samples with contrasting concentrations (low and high concentration samples) at several different ratios allowed assessing the assay accuracy and parallelism with a method that will be reflective of real-world endogenous protein measurements when compared to the recombinant or purified protein source used to calibrate the assays.^19^ The per cent recovery of the observed concentrations when compared to the expected concentrations were used to determine accuracy and parallelism of the assay (80–120% of recovery). Twenty mixed combinations (from four multiple sclerosis samples) showed median recovery of all analytes from 91% to 100%.^19^

The intra- and inter-assay precision, sensitivity and reference ranges for all biomarkers were already reported.^19^ All but one individual biomarker (exception of COL4A1) demonstrated low CV (<20%), and all but two biomarkers (COL4A1 and GH) demonstrated low diurnal variability (<30%).^19^

The Octave-based custom assay panel utilizes the two Uman antibodies (UD1 and UD2, also known as clones 2.1 and 47.3) against NfL that were recently reported.^20^ Furthermore, the assay was calibrated relative to the Quanterix Simoa™ bead-based assay.^20^ Therefore, results for the two assays are consistent in terms of the absolute quantitation output (pg/mL). The correlation coefficient regarding the NfL levels between Simoa assay and the Octave assay was determined to be 0.889. The average per cent difference of the pg/mL reported concentrations between samples measured on both assays was determined to be 4% (median per cent difference = 0%).

### MRI acquisition and analyses

At baseline and follow-up visits, all people with multiple sclerosis underwent MRI examination using the same 3.0T Signa Excite 12 Twin-Speed scanner (GE Healthcare, Milwaukee, WI, USA) and eight-channel head and neck coil. The standard sequences utilized in these analyses were two-dimensional (2D) fluid attenuated inversion recovery (FLAIR), 2D T_1_-weighted spin echo with and without the use of 0.2 mL/kg gadolinium (Gd) contrast acquired 5 min post-injection and high-resolution 3D T_1_-weighted imaging acquired prior to Gd administration. The sequence parameters of the conventional sequences are provided in detail elsewhere.^21^ During the study enrollment (2009–2011), the selected conventional sequences were contemporary at that time. More recent guidelines have recommended the substitution of 2D T2-FLAIR with 3D sequences.^22^ The protocol was kept consistent longitudinally to decrease the potential bias. An additional 2D echo-planar imaging DTI sequence was acquired with echo time (TE) / repetition time (TR) of 90.9/8600 ms, 96 × 96 matrix, flip angle of 90°, echo train length = 1 and in-plane resolution of 3.33 mm × 3.33 mm plane resolution with 3 mm thick non-gapped slices. The *b*-value was 800 s/mm^2^, and 30 volumes with non-collinear diffusion gradients were acquired.

Lesion analysis was performed in a blinded manner with respect to the patient clinical and proteomics status. T2 lesion volume (LV), T1-LV and Gd-LV were obtained using a semi-automated contouring/thresholding technique using Java Image Manipulation version 6.0 (Xinapse Systems Ltd, http://www.xinapse.com/, Essex, UK). Over the follow-up, the changes in T1 and T2-LV were calculated in absolute millilitres (mL). The brain regions of interest (ROIs) of whole brain (WB), white matter (WM), grey matter (GM) and all normalized for the head size were measured using the SIENAX algorithm (FMRIB Software Library, http://www.fmrib.ox.ac.uk/fsl).^23^ Longitudinal change in whole brain volume (WBV) was determined using the SIENA protocol, and measured as per cent brain volume change (PBVC). Direct longitudinal analysis of GM/WM atrophy was determined using SIENAX multi-timepoint algorithm.^24^ A lesion inpainting technique was used to avoid tissue misclassification.^25^ By removing T2-based ROIs, additional maps of normal-appearing brain tissue (NABT) and NAWM were created.

The DTI processing was performed using the FMRIB’s Diffusion ToolBox, which included correction for motion and eddy-current distortion, and scalar maps of FA, and MD of the NABT, NAWM, GM, T2-LV and T1-LV were created.^26^ Generally, higher MD and lower FA levels were interpreted as greater microstructural/axonal CNS pathology in the specific ROI.

### Statistical analyses

All statistical analyses were performed using SPSS version 26.0 (IBM, Armonk, NY, USA). The data distribution was determined using a visual inspection of histograms and Q–Q plots. Additionally, the distribution was determined using the Kolmogorov–Smirnov test for normality. Cross-sectional comparisons were performed using χ^2^ test for categorical variables, Student’s *t*-test for parametric numerical variables, and Mann–Whitney *U* test for non-parametric numerical variables. Variables with overdispersed count data (such as relapses) were analysed using negative binomial regression. Longitudinal analysis was performed using paired non-parametric Wilcoxon test. Correlation data (Spearman’s ranked correlation coefficients) was visualized using heatmaps produced in GraphPad Prism version 8.0 (San Diego, CA, USA). Due to the exploratory nature of the correlations, correction for multiple comparisons was not performed. The correlation analysis was further repeated independently in the relapsing and progressive multiple sclerosis subpopulations. Additionally, multivariable linear regression analyses with step-wise selection were conducted where the MRI-based microstructural outcome was used as the dependent variable and age, sex, BMI and all proteomic measures as independent predictors. All predictors were placed in one step-wise block. For entry into the regression models, the proteomic data were transformed using log(10). Logistic regression models were similarly used if the dependent variable was of categorical nature. Outcomes such as *R*^2^ standardized β and *P*-values were reported. Logistic regression models were also used to predict the presence of Gd-enhancing lesions by the MSDA score. The comparison of longitudinal change in proteomic data and regression *P*-values underwent false discovery rate correction using the Benjamini–Hochberg procedure. Adjusted *P*-values lower than 0.05 were considered statistically significant.

## Results

### Demographic, clinical and MRI characteristics of the study population

The study population consisted of 202 people with multiple sclerosis with demographic and clinical characteristics shown in Table 2. As expected, the progressive people with multiple sclerosis were significantly older, had longer disease duration and had higher EDSS scores both at baseline and follow-up visits (*P* < 0.001 for all). There were no statistical differences in the rate of DP between the relapsing and progressive people with multiple sclerosis (28.6% versus 37.5%, *P* = 0.251). The relapsing people with multiple sclerosis had significantly more relapses over the follow-up period than the progressive group (*P* < 0.001). Over the follow-up, 18 people with clinically isolated syndrome were reclassified as relapsing-remitting multiple sclerosis, and 14 people with relapsing multiple sclerosis were reclassified as prorgressive. While people with relapsing multiple sclerosis were significantly more often treated with natalizumab (16.9% versus 7.4, *P* < 0.001), there were no significant differences between the two groups in terms of overall baseline DMT use, nor DMTs used at the follow-up visit. As expected, the people with progressive multiple sclerosis had significantly greater pathology measured by conventional MRI measures of T2-LV and T1-LV (*P* < 0.001 and *P* = 0.015) and global measures of WBV, white matter volume and grey matter volume (*P* < 0.001). The people with clincially isolated syndrome/relapsing-remitting multiple sclerosis had on average significantly more Gd lesions when compared to the progressive multiple sclerosis group (*P* < 0.001).

**Table 2:** Demographic, clinical and conventional MRI characteristics of the study population

Demographic and clinical characteristics	People with multiple sclerosis (*n* = 202)	Clinically isolated syndrome/relapsing-remitting multiple sclerosis (*n* = 148)	Progressive multiple sclerosis (*n* = 54)	*P*-value
Female, *n* (%)	151 (74.8)	106 (71.6)	45 (83.3)	0.09^a^
Age at baseline, mean (SD)	47.1 (11.1)	44.1 (10.6)	55.3 (7.9)	**<0.001** ^b^
Time of follow-up, mean (SD)	5.4 (0.6)	5.4 (0.6)	5.5 (0.6)	0.732^b^
BMI at baseline, mean (SD)	27.5 (5.8)	27.9 (6.2)	26.5 (4.5)	0.1^b^
Age of disease onset, mean (SD)	32.9 (9.8)	32.6 (9.0)	33.6 (11.8)	0.6^b^
Disease duration at baseline, mean (SD)	13.4 (10.2)	11.1 (8.5)	21.7 (10.5)	**<0**.**001**^b^
EDSS at baseline, median (IQR)	2.5 (1.5–5.0)	1.5 (1.5–2.5)	6.0 (4.0–6.5)	**<0**.**001**^c^
EDSS at follow-up, median (IQR)	3.0 (1.6–6.0)	2.0 (1.5–3.5)	6.5 (4.0–6.5)	**<0**.**001**^c^
EDSS absolute change, mean (SD)	0.4 (0.9)	0.4 (0.9)	0.4 (0.7)	**<0**.**001**^b^
Disability progression, *n* (%)^e^	56 (30.9)	38 (28.6)	18 (37.5)	0.251^a^
Conversion from relapsing-remitting multiple sclerosis to progressive multiple sclerosis, *n* (%)	14 (6.9)	14 (6.9)	-	-
Relapse rate over the follow-up, mean (SD)	0.172 (0.369	0.204 (0.4)	0.09 (0.24)	**<0**.**001**^d^
DMT at baseline, *n* (%)				
IFN-β	85 (42.1)	60 (40.5)	25 (46.3)	0.271^a^
Glatiramer acetate	37 (18.3)	24 (16.2)	13 (24.1)	
Natalizumab	29 (14.4)	25 (16.9)	4 (7.4)	
Off-label DMT	5 (2.5)	3 (2.0)	2 (3.7)	
No DMT	46 (22.8)	36 (24.3)	10 (18.5)	
DMT at follow-up, *n* (%)				
IFN-β	68 (33.7)	52 (35.1)	16 (29.6)	0.797^a^
Glatiramer acetate	45 (22.3)	31 (20.9)	14 (25.9)	
Natalizumab	15 (7.4)	12 (8.1)	3 (5.6)	
Oral DMT	28 (13.9)	22 (14.9)	6 (11.1)	
Off-label DMT	12 (5.9)	8 (5.4)	4 (7.4)	
No DMT	34 (16.8)	23 (15.5)	11 (20.4)	
T2-LV, mean (SD)	13.5 (16.7)	10.3 (14.09)	22.2 (20.1)	**<0**.**001**^b^
T1-LV, mean (SD)	3.0 (7.2)	2.2 (6.44)	5.5 (8.6)	**0**.**015**^b^
Gd-LN, mean (SD)	0.05 (3.2)	0.7 (3.7)	0.04 (0.2)	**<0**.**001**^c^
Gd-LV, mean (SD)	0.07 (0.4)	0.1 (0.48)	0.01 (0.03)	0.194^b^
WBV, mean (SD)	1466.4 (94.3)	1490.2 (87.7)	1401.7 (80.8)	**<0**.**001**^b^
WMV, mean (SD)	725.9 (62.2)	738.7 (62.1)	689.8 (46.7)	**<0**.**001**^b^
GMV, mean (SD)	740.8 (63.9)	751.4 (65.9)	711.9 (48.1)	**<0**.**001**^b^

Thirteen (13) people with clinically isolated syndrome or relapsing-remitting multiple sclerosis transitioned into progressive multiple sclerosis over the follow-up. All volumes are presented in millilitres. Parametric data are shown as mean (standard deviation), whereas non-parametric data are shown as median (interquartile range). *P*-values lower than 0.05 were considered statistically significant and shown in bold.

BMI, body mass index; EDSS, Expanded Disability Status Scale; DMT, disease-modifying therapy; IFN, interferon; SD, standard deviation; IQR, interquartile range; LV, lesion volume; LN, lesion number; WBV, whole brain volume; WMV, white matter volume; GMV, grey matter volume.

a The specific comparisons were performed using χ^2^ test.

b The specific comparisons were performed using Student’s *t*-test.

c The specific comparisons were performed using Mann–Whitney *U* test.

d The specific comparisons were performed using negative binomial regression.

e Disability progression was available for 181 out of 202 people with multiple sclerosis due to missing EDSS values at either baseline or follow-up visit.

People with progressive multiple sclerosis also had greater age-adjusted microstructural (axonal) CNS pathology as evidenced by greater MD values of all MRI structural and lesion-based regions (NABT, *P* < 0.001; NAWM, *P* < 0.001; GM, *P* = 0.005; T2-LV, *P* = 0.011; T1-LV, *P* = 0.003). Similar findings were seen with the FA measure, where people with progressive multiple sclerosis had lower FA of the NABT (*P* = 0.003), NAWM (*P* = 0.045) and T1-LV FA (*P* = 0.007). All DTI-based measures for the people with multiple sclerosis and the multiple sclerosis subtypes are shown in Table 3.

**Table 3:** Diffusion tensor imaging outcomes at baseline in the study population

DTI-based outcomes	People with multiple sclerosis (*n* = 202)	Clinically isolated syndromeIS/relapsing-remitting multiple sclerosis (*n* = 148)	Progressive multiple sclerosis (*n* = 54)	*P*-value	Age-adjusted *P*-value
NABT MD	1.1 (0.09)	1.08 (0.08)	1.16 (0.09)	**<0.001**	<**0**.**001**
NAWM MD	0.95 (0.08)	0.93 (0.07)	1.0 (0.1)	**<0**.**001**	**<0**.**001**
GM MD	1.24 (0.11)	1.22 (0.1)	1.31 (0.1)	**<0**.**001**	**0**.**005**
T2-LV MD	1.12 (0.13)	1.09 (0.12)	1.2 (0.14)	**<0**.**001**	**0**.**011**
T1-LV MD	1.31 (0.21)	1.25 (0.2)	1.43 (0.17)	**<0**.**001**	**0**.**003**
NABT FA	0.25 (0.02)	0.25 (0.19)	0.24 (0.018)	0.05	**0**.**03**
NAWM FA	0.33 (0.03)	0.33 (0.032)	0.32 (0.03)	**0**.**011**	**0**.**045**
GM FA	0.17 (0.01)	0.17 (0.015)	0.17 (0.013)	0.676	0.945
T2-LV FA	0.32 (0.04)	0.32 (0.042)	0.30 (0.037)	**0**.**006**	0.191
T1-LV FA	0.26 (0.05)	0.27 (0.053)	0.24 (0.038)	**<0**.**001**	**0**.**007**

The disease classification is based on the baseline phenotype. All comparisons were performed using Student’s *t*-test. Mean diffusivity (MD) are shown in mm^2^/s as multiplied by a factor of 1000. FA is a unitless measure. Age-adjusted *P*-value was derived using analysis of covariance (ANCOVA). *P*-values lower than 0.05 were considered statistically significant and shown in bold.

DTI, diffusion tensor imaging; MD, mean diffusivity; FA, fractional anisotropy; GM, grey matter; NABT, normal-appearing brain tissue; NAWM, normal-appearing white matter; LV, lesion volume.

### Proteomic data and disability progression in the study population

In total, 143 people with multiple sclerosis (104 relapsing and 39 progressive multiple sclerosis) had paired serum samples at both baseline and follow-up visits. The baseline, follow-up and longitudinal changes in each of the 21 proteomic biomarkers (shown as median and interquartile range) are shown in Table 4. The same analyses performed separately independently in relapsing and progressive multiple sclerosis groups are shown in Supplemental Table 1. Over the follow-up, people with multiple sclerosis had significant increase in CCL20 (9.7 pg/mL versus 15.1 pg/mL, *P* = 0.001) and NfL levels (10.5 pg/mL versus 11.5 pg/mL, *P* = 0.003). In an age-adjusted binary logistic model, the classification of relapsing-remitting and progressive multiple sclerosis was predicted by baseline GFAP (*P* = 0.018), MOG (*P* = 0.003) and VCAN (*P* = 0.029) levels with 82.1% accuracy (Nagelkerke *R*^2^ = 0.483).

**Table 4:** Proteomic panel in the study population at baseline and over the follow-up

Proteomic data	Baseline median (IQR)	Follow-up median (IQR)	Median (IQR) % change	Expected change in worsening	143 sample paired test *P*-value
APLP1 in ng/mL	11.9 (9.7–14.6)	12.7 (10.4–14.6)	2.4 (−16.2–25.8)	Unknown	0.243
CCL20	9.7 (6–22.4)	15.1 (8–37.5)	63.5 (−26.9–207)	Increase	0.001*
CD6	139.3 (103.7–188.9)	142.1 (110.1–182)	5.9 (−26.2–36.6)	Increase	0.867
CDCP1	106.6 (78.5–140.2)	108 (79.8–159.1)	13.5 (−17.6–45)	Increase	**0**.**008**
CNTN2 in ng/mL	1.8 (1.4–2.5)	1.9 (1.5–2.8)	8.8 (−13–29.8)	Increase	**0**.**016**
COL4A1 in ng/mL	1.3 (0.9–2.1)	1.2 (0.9–1.7)	−3.6 (−32.9–25.5)	Unknown	0.081
CXCL13	50.4 (40.2–69.9)	51.5 (42.4–70.2)	4.5 (−22.9–37.1)	Increase	0.555
CXCL9	54.4 (37.8–89.6)	64.8 (38.2–99.5)	7.8 (−23.8–54.1)	Increase	0.089
FLRT2	114 (95.2–137.5)	117.5 (98.6–136.3)	2.6 (−15.1–16.3)	Increase	0.562
GFAP	122.2 (86.6–164.2)	128.8 (93.1–176.1)	6.7 (−14.6–38.8)	Increase	**0**.**041**
GH	193.4 (69.3–791.4)	164.9 (68–503.1)	−31.7 (−74.4–152.1)	Decrease	0.078
IL-12B	115.4 (77.5–183)	111.5 (77.6–172.6)	0 (−24.6–27)	Increase	0.568
MOG	28.4 (23.2–36)	31.3 (24.1–37.8)	8.8 (−15.5–34.3)	Bi-directional	**0**.**02**
NfL	10.5 (7.7–14.6)	11.5 (8.6–16.1)	10.2 (−14.3–39.2)	Increase	**0**.003*
OPG	811.4 (656.9–948.2)	822.5 (664–1046.9)	3.2 (−14.6–27.4)	Increase	0.089
OPN in ng/mL	20.3 (15.0–25.1)	21.3 (16.6–27.3)	8.5 (−19.8–45.6)	Increase	**0**.**025**
PRTG	128 (110.1–152.1)	133.7 (112.3–153.5)	0.6 (−12–13.2)	Decrease	0.913
SERPINA9	52.7 (35–81.9)	54.9 (36.3–78.9)	−4.2 (−33.1–38.7)	Unknown	0.933
TNFRSF10A	6 (4.9–7.5)	6.4 (5–7.8)	3.6 (−12.9–29.1)	Increase	0.145
TNFSF13B in ng/mL	4.9 (4.2–6.2)	5.1 (4.2–6.2)	0.3 (−15.7–18.5)	Increase	0.939
VCAN	450.8 (388–533.4)	466.9 (391.7–531.5)	0.9 (−14.5–16.5)	Bi-directional	0.88

All measures are shown in picogram in millilitre (pg/mL) unless specified in the table. The change in proteomic data was calculated based on 143 available paired samples. All comparisons were performed using paired non-parametric Wilcoxon test. *P*-values lower than 0.05 were considered statistically significant and shown in bold. Only measures labelled with asterisks (*) survived Benjamini–Hochberg correction for false discovery rate (FDR). The expected changes in worsening are based on literature reports in multiple sclerosis, other neurological diseases or in system inflammation processes.

IQR, interquartile range; APLP1, amyloid beta precursor like protein 1; CCL20, C-C motif chemokine ligand 20; CD6, T-cell differentiation antigen CD6; CDCP1, CUB domain-containing protein 1; CNTN2, contactin 2; COL4A1, collagen type IV alpha 1; CXCL13, chemokine ligand 13; CXCL9, chemokine ligand 9; FLRT2, fibronectin leucine-rich transmembrane protein 2; GFAP, glial fibrillary acidic protein; GH, growth hormone; IL-12B, interleukin 12 subunit B; MOG, myelin-oligodendrocyte glycoprotein; NfL, neurofilament light chain; OPG, osteoprotegerin; OPN, osteopontin; PRTG, protogenin precursor; SERPINA9, serpin family A member A; TNFRSF10A, tumour necrotic factor receptor superfamily member 10a; TNFSF13b, tumour necrotic factor superfamily member 13b; VCAN, versican.

With age, sex, BMI and all proteomic biomarkers included in the regression model, the DP over the follow-up was predicted by higher baseline GFAP (Exp(B) = 8.647, *P* = 0.004). The final model had predictive accuracy for having DP over the follow-up of 72.9% (Nagelkerke *R*^2^ = 0.069). Out of the 56 people with multiple sclerosis with DP, 25 (44.6%) had experienced a relapse over the follow-up, and 31 (55.4%) progressed without any relapse recorded. These two groups were best predicted by the higher baseline serum neurofilament light chain (sNfL) levels (Exp(B) = 0.031, *P* = 0.01) in the non-relapsing DP people with multiple sclerosis when compared to people with multiple sclerosis that had concurrent DP and relapses over the follow-up (accuracy of 74.1, Nagelkerke *R*^2^ = 0.187). With both conventional measures and proteomic data added as predictors, only baseline T2-LV was significantly associated with DP (*P* = 0.001). The differences in proteomic data based on the DMT treatment at baseline and between changes in therapy are shown in Supplemental Tables 2 and 3. People with multiple sclerosis treated with high-potency DMT (natalizumab) had the lowest inflammation-based protein levels when compared to people with multiple sclerosis treated with IFN-β, glatiramer acetate or not treated with any DMT. In particular, people with multiple sclerosis on natalizumab had lower CD6, CDCP1, CXCL13, CXCL9, NfL, TNFRSF10a, TNFSF13B and VCAN (*P* < 0.036). There were no statistical differences in the change of proteomic data over the follow-up between the DMT groups.

### Relationship between conventional MRI measures over the follow-up and baseline proteomic data

In our sample, the presence of Gd-enhancing lesions was successfully predicted by a higher baseline MSDA score (Exp(B) = 1.565, *P* < 0.001) with 85.7% accuracy (Nagelkerke *R*^2^ = 0.159). When all proteomic biomarkers were utilized as a part of the model, a higher number of Gd-enhancing lesions were predicted by greater NfL levels (standardized β = 0.295, *P* < 0.001) and younger age (standardized β = −0.232, *P* = 0.003). The relationships between baseline proteomic measures and changes in conventional MRI measures over the follow-up are shown in Supplemental Table 4. The extent of absolute T2-LV increase over the follow-up was significantly associated with higher IL-12B levels (standardized β = 0.299, *P* < 0.001). No baseline proteomic measure was able to predict the amount of T1-LV development over the follow-up. The PBVC over the 5-year follow-up was predicted by higher baseline GFAP (standardized β = −0.296, *P* = 0.001), higher NfL (standardized β = −0.266, *P* = 0.003) and lower levels of baseline PRTG (standardized β = 0.207, *P* = 0.009) and MOG (standardized β = 0.26, *P* = 0.010). The rate of GM atrophy was associated with higher baseline NfL levels (standardized β = −0.197, *P* = 0.016), lower PRTG levels (standardized β = 0.273, *P* = 0.003) and higher OPN levels (standardized β = −0.329, *P* < 0.001). The rate of WM atrophy was not significantly predicted by any proteomic biomarker. In particular, the baseline GFAP levels were not predictive of the rate of WM or GM atrophy over the follow-up period.

### Cross-sectional and longitudinal relationship between microstructural/axonal CNS pathology and proteomic data

**Figure 1:**
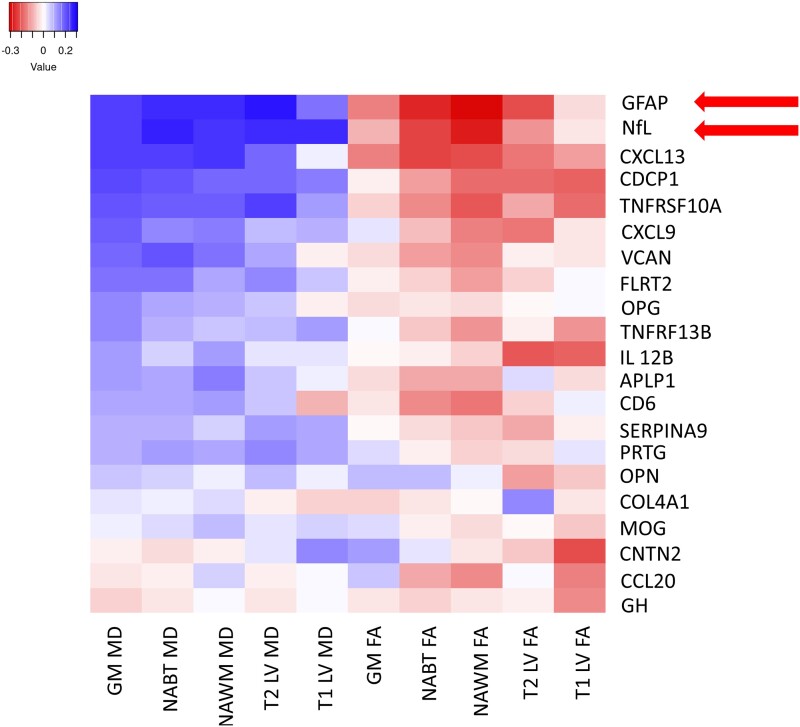
**Cross-sectional correlation matrix (heatmap) between follow-up proteomic data and follow-up diffusion tensor imaging outcomes in people with multiple sclerosis.** Correlation data (Spearman’s ranked correlation coefficients) was performed in 202 people with multiple sclerosis. The magnitude of the correlation coefficient is shown in the legend. Red squared demonstrates positive correlation coefficients, and blue squares demonstrate negative correlation coefficients. Red arrows indicate biomarkers with significant correlations with DTI-based measures. MD, mean diffusivity; FA, fractional anisotropy; GM, grey matter; NABT, normal-appearing brain tissue; NAWM, normal-appearing white matter; LV, lesion volume; APLP1, amyloid beta precursor like protein 1; CCL20, C-C motif chemokine ligand 20; CD6, T-cell differentiation antigen CD6; CDCP1, CUB domain-containing protein 1; CNTN2, contactin 2; COL4A1, collagen type IV alpha 1; CXCL13, chemokine ligand 13; CXCL9, chemokine ligand 9; FLRT2, fibronectin leucine-rich transmembrane protein 2; GFAP, glial fibrillary acidic protein; GH, growth hormone; IL-12B, interleukin 12 subunit B; MOG, myelin-oligodendrocyte glycoprotein; NfL, neurofilament light chain; OPG, osteoprotegerin; OPN, osteopontin; PRTG, protogenin precursor; SERPINA9, serpin family A member A; TNFRSF10A, tumour necrotic factor receptor superfamily member 10a; TNFSF13b, tumour necrotic factor superfamily member 13b; VCAN, versican.

**Figure 2:**
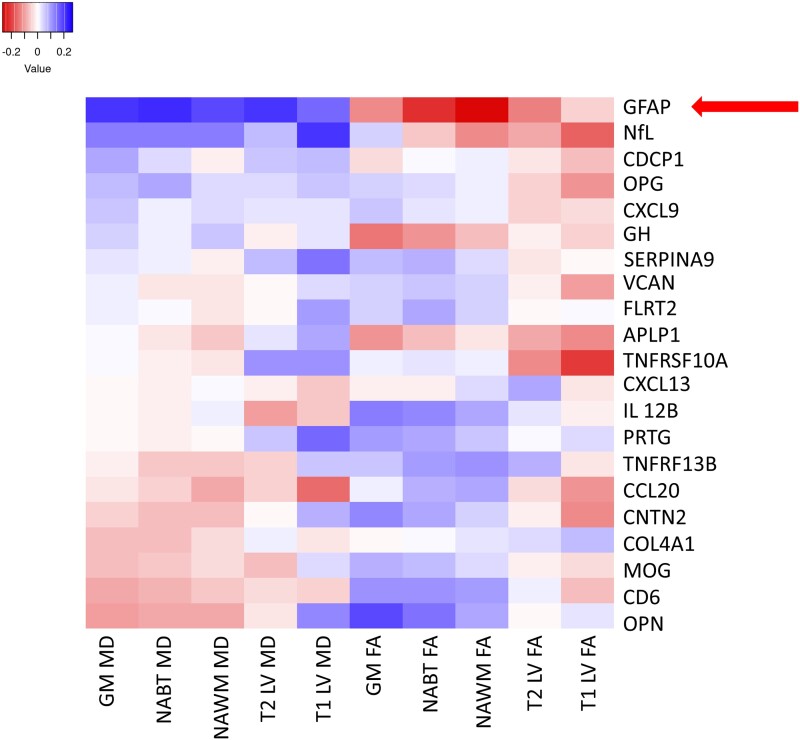
**Longitudinal correlation matrix (heatmap) between baseline proteomic data and follow-up diffusion tensor imaging outcomes in people with multiple sclerosis.** Correlation data (Spearman’s ranked correlation coefficients) were performed in 181 people with multiple sclerosis with available baseline proteomic data and follow-up DTI data. The magnitude of the correlation coefficient is shown in the legend. Red squared demonstrates positive correlation coefficients, and blue squares demonstrate negative correlation coefficients. Red arrows indicate biomarkers with significant correlations with DTI-based measures. MD, mean diffusivity; FA, fractional anisotropy; GM, grey matter; NABT, normal-appearing brain tissue; NAWM, normal-appearing white matter; LV, lesion volume; APLP1, amyloid beta precursor like protein 1; CCL20, C-C motif chemokine ligand 20; CD6, T-cell differentiation antigen CD6; CDCP1, CUB domain-containing protein 1; CNTN2, contactin 2; COL4A1, collagen type IV alpha 1; CXCL13, chemokine ligand 13; CXCL9, chemokine ligand 9; FLRT2, fibronectin leucine-rich transmembrane protein 2; GFAP, glial fibrillary acidic protein; GH, growth hormone; IL-12B, interleukin 12 subunit B; MOG, myelin-oligodendrocyte glycoprotein; NfL, neurofilament light chain; OPG, osteoprotegerin; OPN, osteopontin; PRTG, protogenin precursor; SERPINA9, serpin family A member A; TNFRSF10A, tumour necrotic factor receptor superfamily member 10a; TNFSF13b, tumour necrotic factor superfamily member 13b; VCAN, versican.

Exploratory cross-sectional and longitudinal (associations between baseline proteomic and follow-up MRI data) correlation analyses between proteomic data and DTI-based outcomes are shown in Figs 1 and 2, respectively. At baseline, greater microstructural/axonal pathology was associated with greater GFAP levels (*P* < 0.001 for all structural and lesion ROIs except for T1-LV FA). To a lesser extent (albeit still statistically significant), greater NfL levels were cross-sectionally associated with greater axonal pathology as measured by all but one MD measure (*P* < 0.024, T1-LV MD was the only non-significant correlation). Contrarily, only higher baseline GFAP levels were associated with greater axonal pathology at follow-up.

Cross-sectionally at baseline, the MSDA score and disease pathway scores were not associated with any DTI-based measure (data not shown). No significant correlations between baseline MSDA and disease pathway scores and follow-up DTI-based measures were noted (data not shown). These findings were expected since the algorithm utilized to create the MSDA and pathway scores was originally trained and validated relative to the presence of MRI-based Gd-enhancing lesions at the same time of sample acquisition.

### Regression analyses between proteomic data and microstructural/axonal CNS pathology at baseline and follow-up

In order to pinpoint the biomarkers predictive of axonal pathology, regression models for each region were performed. After adjusting for potential significant effects by age, sex and BMI, NABT FA variance was additionally explained by GFAP (standardized β = −0.265, *P* = 0.001) and OPN (standardized β = 0.237, *P* = 0.003). NAWM FA was similarly explained by both GFAP (standardized β = −0.407, *P* < 0.001) and OPN (standardized β = 0.247, *P* = 0.002). Additional cross-sectional effects of OPN, APLP1, COL4A1, FLRT2 and PRTG on cross-sectional GM, T1-LV and T2-LV FA measures were noted and shown in Table 5. Similar findings were seen with MD measures, where GFAP was the strongest significant predictor of NABT MD (standardized β = 0.404, *P* < 0.001), NAWM MD (standardized β = 0.494, *P* < 0.001), GM MD (standardized β = 0.364, *P* < 0.001) and T1-LV MD (standardized β = 0.237, *P* = 0.023).

**Table 5:** Linear step-wise regression determining cross-sectional associations between baseline proteomics and microstructural DTI-based measures in people with multiple sclerosis at the baseline visit

Fractional anisotropy (FA)	Predictors	*R* ^2^	Standardized β	*P*-value	Mean diffusivity (MD)	Predictors	*R* ^2^	Standardized β	*P*-value
NABT FA	Sex	0.097	0.259	**0.001**	NABT MD	GFAP	0.14	0.404	**<0**.**001**
	BMI	0.174	0.223	**0**.**005**		MOG	0.216	−0.538	**<0**.**001**
	GFAP	0.214	−0.265	**0**.**001**		CNTN2	0.271	0.23	**0**.**01**
	OPN	0.266	0.237	**0**.**003**		BMI	0.314	−0.265	**0**.**001**
NAWM FA	GFAP	0.136	−0.407	**<0**.**001**		OPG	0.344	0.196	**0**.**012**
	OPN	0.213	0.247	**0**.**002**		Age	0.369	0.182	**0**.**026**
	Sex	0.247	0.19	**0**.**016**	NAWM MD	GFAP	0.136	0.494	**<0**.**001**
GM FA	OPN	0.047	0.307	**0**.**001**		MOG	0.19	−0.371	**<0**.**001**
	APLP1	0.087	−0.22	**0**.**018**		CNTN2	0.219	0.205	**0**.**028**
T2-LV FA	Age	0.067	−0.357	**<0**.**001**	GM MD	GFAP	0.132	0.364	**<0**.**001**
	PRTG	0.111	0.324	**0**.**001**		MOG	0.207	−0.556	**<0**.**001**
	COL4A1	0.159	−0.242	**0**.**008**		CNTN2	0.27	0.227	**0**.**009**
T1-LV FA	Age	0.036	−0.244	**0**.**013**		BMI	0.321	−0.3	**<0**.**001**
	FLRT2	0.091	0.24	**0**.**014**		Age	0.363	0.246	**0**.**002**
						OPG	0.408	0.23	**0**.**002**
					T2-LV MD	Age	0.138	0.371	**<0**.**001**
					T1-LV MD	Age	0.139	0.363	**<0**.**001**
						MOG	0.195	−0.465	**<0**.**001**
						GFAP	0.251	0.237	**0**.**023**
						SERPINA9	0.289	0.228	**0**.**012**
						IL-12B	0.32	−0.202	**0**.**022**
						OPN	0.348	0.192	**0**.**041**

Step-wise regression model was utilized where the DTI-based measures were considered as a dependent variable, and all proteomic data were added as independent predictors. All proteomic data were logarithmically transformed log(10). In addition to the proteomic data, the age, sex and BMI of the people with multiple sclerosis were also included. *P*-values lower than 0.05 were considered as statistically significant and shown in bold.

BMI, body mass index; GFAP, glial fibrillary acidic protein; OPN, osteopontin; COL4A1, collagen type IV alpha 1; PRTG, protogenin precursor; CNTN2, contactin 2; OPG, osteoprotegerin; SERPINA9, serpin family A member A; IL-12B, interleukin 12 subunit B; MOG, myelin-oligodendrocyte glycoprotein; DTI, diffusion tensor imaging; MD, mean diffusivity; FA, fractional anisotropy; GM, grey matter; NABT, normal-appearing brain tissue; NAWM, normal-appearing white matter; LV, lesion volume.

Greater axonal pathology in all tissue-specific brain structures was further explained by lower MOG levels (standardized β = −0.538, *P* < 0.001 for NABT MD, standardized β = −0.371, *P* < 0.001 for NAWM MD and standardized β = −0.556, *P* < 0.001 for GM MD, respectably). An additional reoccurring proteomic predictor was CNTN2 for all brain-specific volumes (*P* < 0.028) and OPG (for NABT MD and GM MD). Lastly, the variance in T2-LV MD was further explained by SERPINA9, IL-12B and OPN levels (data shown in Table 5). The cross-sectional analysis for follow-up proteomic measures and follow-up DTI-based outcomes is shown in Supplemental Table 5.

At follow-up, results were similar to those seen at baseline, with higher GFAP, lower MOG and higher NfL levels showing the most consistent relationship with various DTI outcomes.

### Regression analyses between proteomic data and microstructural/axonal CNS pathology over the follow-up

The ability of baseline proteomic data to predict future axonal pathology is shown in Table 6. After adjusting for age, sex and BMI effects, higher baseline GFAP and lower baseline MOG levels were predictors of both future lower NABT FA (standardized β = −0.397, *P* < 0.001 and standardized β = 0.191, *P* = 0.01, respectively) and future lower NAWM FA (standardized β = −0.466, *P* < 0.001 and standardized β = 0.221, *P* = 0.025, respectively). GM FA was predicted by OPN (standardized β = 0.133, *P* = 0.021), APLP1 (standardized β = −0.245, *P* = 0.011) and CD6 (standardized β = 0.192, *P* = 0.029). Baseline GFAP levels were predictors of future axonal pathology 5 years later in all brain regions, including NABT MD (standardized β = 0.327, *P* = 0.001), NAWM MD (standardized β = 0.214, *P* = 0.012), GM MD (standardized β = 0.346, *P* = 0.001) and T2-LV MD (standardized β = 0.416, *P* < 0.001). Lower MOG levels were also predictors of greater axonal pathology measured as NABT MD (standardized β = −0.224, *P* = 0.027), GM MD (standardized β = −0.256, *P* = 0.011) and T2-LV MD (standardized β = −0.332, *P* = 0.001). Future T1-LV MD was predicted by baseline NfL levels (standardized β = 0.299, *P* = 0.003).

**Table 6:** Linear step-wise regression determining associations between baseline proteomics and future microstructural DTI-based outcomes (at follow-up) in people with multiple sclerosis

Fractional anisotropy (FA)	Predictors	*R* ^2^	standardized β	*P*-value	Mean diffusivity (MD)	Predictors	*R* ^2^	standardized β	*P*-value
NABT FA	GFAP	0.113	−0.397	**<0.001**	NABT MD	GFAP	0.041	0.327	**0**.**001**
	BMI	0.156	0.233	**0**.**005**		MOG	0.076	−0.224	**0**.**027**
	MOG	0.191	0.253	**0**.**01**	NAWM MD	GFAP	0.046	0.214	**0**.**012**
	Sex	0.231	0.203	**0**.**013**	GM MD	GFAP	0.041	0.346	**0**.**001**
NAWM FA	GFAP	0.146	−0.466	**<0**.**001**		MOG	0.087	−0.256	**0**.**011**
	MOG	0.178	0.221	**0**.**025**	T2-LV MD	GFAP	0.072	0.416	**<0**.**001**
	BMI	0.205	0.168	**0**.**044**		MOG	0.118	−0.332	**0**.**001**
GM FA	BMI	0.053	0.198	**0**.**021**		TNFRSF10a	0.152	0.196	**0**.**025**
	Sex	0.103	0.154	**0**.**07**	T1-LV MD	NfL	0.09	0.299	**0**.**003**
	OPN	0.133	0.205	**0**.**021**					
	APLP1	0.161	−0.245	**0**.**011**					
	CD6	0.193	0.192	**0**.**029**					
T2-LV FA	Age	0.082	−0.287	**0**.**001**					
T1-LV FA	Sex	0.079	0.283	**0**.**003**					

Step-wise regression model was utilized where the DTI-based measures were considered as a dependent variable, and all proteomic data were added as independent predictors. In addition to the proteomic data, the age, sex and BMI of the people with multiple sclerosis were also included. *P*-values lower than 0.05 were considered statistically significant and shown in bold.

GFAP, glial fibrillary acidic protein; MOG, myelin-oligodendrocyte glycoprotein; IL-12B, interleukin-12 subunit B; FLRT2, fibronectin leucine-rich transmembrane protein 2; TNFRSF10a, tumour necrotic factor receptor superfamily member 10a; NfL, neurofilament light chain; DTI, diffusion tensor imaging; MD, mean diffusivity; FA, fractional anisotropy; GM, grey matter; NABT, normal-appearing brain tissue; NAWM, normal-appearing white matter; LV, lesion volume.

The longitudinal trajectories in microstructural properties were further calculated as a percentage change over the follow-up. No consistent pattern of baseline proteomic predictors was associated with the extent of follow-up DTI-based change. In particular, worsening per cent change in NAWM FA was predicted by higher baseline CCL20 (standardized β = 0.228, *P* = 0.009) and lower OPN (standardized β = −0.321, *P* = 0.001). Similarly, worsening T2-LV FA changes were predicted by baseline CXCL13 (standardized β = 0.331, *P* < 0.001) and PRTG (standardized β = −0.236, *P* = 0.011).

Lastly, the correlations between baseline proteomic data and follow-up DTI-based measures are shown in Supplemental Fig. 1. In both the multiple sclerosis subgroups, greater GFAP levels remained as the only significantly associated biomarker with more severe DTI measures.

## Discussion

The findings of this study are multifold. Firstly, higher baseline serum GFAP levels were predictive of future DP. Secondly, among several serum proteins, GFAP, MOG and NfL showed independent and consistent relationships with the severity of microstructural CNS axonal pathology. On the other hand, higher GFAP levels were not associated with volumetric WM or GM changes. These findings suggest that the use of a multivariate proteomic assay has a considerably greater clinical value when compared to the use of a single proteomic measure. In addition to their cross-sectional relationship, higher GFAP and lower MOG serum levels were predictive of future microstructural alterations.

Previous conference reports have described the validity and utility of the multivariate proteomic assay’s ability to predict MSDA.^27^ When compared to univariate biomarkers, the MSDA score was significantly superior in predicting clinical and/or MRI-based disease activity of Gd-enhancing lesions and new enlarging T2 lesions.^28^ In this MSDA composite, NfL repeatedly presents as the most important contributor. Similar findings were seen in our analysis, where MSDA was further validated and was able to predict the presence of Gd-enhancing lesions. Moreover, the number of Gd-enhancing lesions in our sample was also predicted by the NfL levels. That said, distinct differences between biomarkers associated with acute inflammatory activity and microstructural pathology are observed, with NfL and GFAP as the main proxy measures for the respective pathophysiological mechanisms. In comparison to NfL, a study has shown that acute clinical relapses and the presence of Gd-enhancing lesions do not result in a significant increase in serum GFAP levels.^29^ Although a high-potency DMT such as natalizumab leads to a multifold decrease of NfL, the GFAP levels remain relatively stable and unaffected.^30^ The same findings were also seen in our analysis where DMTs significantly decreased the serum levels of NfL and other inflammatory proteins (e.g. CD6, CDCP1, CXCL13, CXCL9 and TNFRSF10A), but did not affect GFAP levels (Supplement Table 2).

The pathophysiological processes in the later progressive phase of multiple sclerosis heavily implicate worsening astrocytopathology and resident microglia activation.^31^ Along those lines, only GFAP and none of the inflammatory proteins were able to predict DP over the 5-year follow-up period within our relatively older study population. Serum GFAP was also the best differentiator between relapsing and progressive multiple sclerosis groups, a finding that is consistently featured in the recent literature.^32,33^ The utility of GFAP as a predictor of subsequent progression in older and non-active people with multiple sclerosis has been recently demonstrated in similar cohorts as well.^34,35^ For example, the progression over 7.6 years in non-active people with multiple sclerosis with an average baseline of EDSS of 4.0 was only predicted by higher serum GFAP levels and not associated with sNfL levels.^34^ This association was particularly stronger in a cohort of people with multiple sclerosis with initially low sNfL levels.^34^ The utility of GFAP levels to provide additional value in predicting DP even after adjusting for sNfL effect was also showcased within a large multicentre Swiss-based study that recruited 355 people with multiple sclerosis with a similar average age between 42 and 44 years old.^35^ This study also demonstrated a significant association of serum GFAP levels only with accelerated GM atrophy and not with any WM-based changes.^35^ Such findings indirectly validate our results of GFAP being one of the most sensitive to pathological changes in relatively older, non-active and progressive populations.

One of the main objectives of this study was to investigate which proteomic biomarkers were predictive of CNS axonal pathology development, as determined by DTI. Although we collected additional conventional MRI measures at baseline and follow-up (inflammatory lesions and measures of brain atrophy), our goal in this study was to focus specifically on proteomic correlates of axonal multiple sclerosis pathology. The predictive value of proteomics biomarkers with respect to conventional MRI outcomes (lesions and brain atrophy) collected in this study will be further reported. Only one previous study examined the cross-sectional relationship specifically between GFAP and DTI-based measures and showed similar findings.^36^ In that study, higher serum GFAP levels measured in 62 people with multiple sclerosis were significantly associated with lower NABT/NAWM FA and higher NAWM MD values.^36^ We corroborate their findings and further extend them with mid-term longitudinal data. These unique findings have far-reaching translational implications, particularly since they corroborate basic neuropathological and pathophysiological mechanisms related to the role of astrocytes in long-term multiple sclerosis progression.^37^ The edge of chronic active lesions, a pathological hallmark of progressive multiple sclerosis, is abundant with activated astrocytes when compared to unaffected regions (astrocytes being 19% of all cells when compared to only 6% astrocytes in the NAWM).^38^ Previously affected areas devoid of acute inflammation also retain the astrocytic hypercellularity, and the brain tissue does not return to a ‘healthy’ cell composition.^38^ Most importantly, post-mortem studies demonstrate widespread microglial nodules within the NAWM that are directly linked to diffuse axonal (Wallerian) degeneration.^39^ These changes are histopathologically visualized as microglial nodules along injured and degenerating axons, particularly abundant in the vicinity of axonal (NPY-Y1R^+^) fragments.^39^ Based on this spatial relationship, the formation of astrogliosis in non-lesioned WM may be an outcome sequela of Wallerian degeneration.^39^ Our study validates these mechanisms using two *in vivo* and non-invasive techniques. That said, the microstructural changes in the NAWM may be diffused and unrelated to focal demyelinating lesions.^40^ In order to understand which biomarkers may be indicative of demyelination-induced versus independent microstructural pathology, future analysis could focus on comparing the biomarker relationships with specific tracts that are either affected or not by a demyelinating lesion. Moreover, tract-based spatial statistics could supplement the longitudinal analysis and better determine the relationship between proteomic data and absolute/per cent-based DTI changes, which will be subject of the future work.

The concept of serum GFAP levels as an *in vivo* measure of astrocytic pathology has already been proven successful in large Phase 3 trials related to neuroinflammatory disorders and traumatic brain injury. In the N-MOmentum trial, higher baseline GFAP levels were associated with future neuroinflammatory attacks in neuromyelitis optica spectrum disorder (NMOSD) and responsive to treatment.^41^ Given that NMOSD pathophysiology has clear astroglial involvement, the GFAP biomarker is well-suited as a study outcome biomarker. Moreover, the FDA has already approved the use of commercial GFAP assays for screening of brain trauma/concussions, and similar proteomic assays (e.g. serum NfL levels) are currently being reviewed for their use in multiple sclerosis disease monitoring. That said, our results argue for use of a multivariate proteomic assay when compared to a single serum measure.

MOG is a mammalian-exclusive protein of the CNS that is present on most external lamina of the myelin sheet and on the surface of myelinating oligodendrocytes.^42^ MOG represents only a very small portion of the total myelin protein, with studies estimating it between 0.01% and 0.05%.^43,44^ While its function is not fully established, studies suggest that MOG protein is responsible for compaction and maintenance of the myelin structure and as a mediator for the transfer of extracellular information into the oligodendrocytes.^45^ MOG has been recognized as a dominant tolerogen for CNS myelin.^46^ Artificial activation of dendritic cells in the presence of MOG particles results in the induction of an anti-inflammatory state and production of IL-10.^47^ In a confirmatory experiment, the removal of the protective MOG interaction results in activation of the inflammasome.^47^

Due to the inconsistent presence of proteins such as contactin-2, osteopontin, osteoprotegerin and CD6 as predictors of DTI-based alterations, their clinical relevance should be carefully interpreted. Although these proteomic biomarkers survived the multiple comparison corrections, their significance was not reproduced in other highly correlated MRI outcomes. Further replication with different multiple sclerosis samples and in different research settings should confirm these findings. Our findings of an association between higher CNTN2 levels with greater axonal pathology have been previously documented as well.^48^ CNTN2 levels have been correlated with the level of NfL, and CNTN2 can predict the longitudinal decline of cortical volume.^48^ Moreover, CNTN2 has been previously implicated as a potential autoantigen in multiple sclerosis with a particular presentation of increased GM pathology.^49^ Similar interpretations are applicable to the OPN findings, where higher OPN levels were associated with enlargement of the lateral ventricles and GM atrophy.^50^ The involvement of CD6 and IL-12B has been previously noted in the literature.^51^ CD6 levels were detectable in up to 22.2% of relapsing-remitting multiple sclerosis as compared to none in controls, and these levels were correlated with IL-12B.^51^

One specific limitation of this analysis is the lack of proteomic data in a healthy control population. Excluding the sNfL data present in the literature, cut-off values and reference intervals of the remaining proteomic biomarkers are currently unknown. Future studies should determine the normal change of these proteomics biomarkers within a healthy control group. The currently utilized 202 people with multiple sclerosis satisfied the common regression rule of 10 patients per predictor used. However, this sample size was insufficient for performing similar multiple sclerosis subtype analyses, and only 143 people with multiple sclerosis had paired longitudinal data. Future studies should determine whether there are differential proteomic biomarkers that are better predictors of cross-sectional and future axonal changes in relapsing and progressive multiple sclerosis groups, respectively. Multiple recent findings suggest that serum protein concentrations are influenced by non-pathology-related factors such as total blood volume (as a function of concentration dilution) and by the glomerular filtration rate (GFR) as means of NfL or other protein clearance.^52,53^ For example, diabetic patients with GFR lower than 60 mL/min/1.79m^2^ had eight times greater serum NfL levels when compared to controls with normal GFR.^54^ As a legacy cohort originally recruited in years 2009–2012, another limitation of the study was that our people with multiple sclerosis were treated with relatively older, less varied and less potent DMTs. Outside of the significantly greater use of natalizumab in the people with clinicaly isolated syndrome/relapsing-remitting multiple sclerosis, both groups received similar DMT protocol with the majority of people with progressive multiple sclerosis continuing their IFN-β and glatiramer acetate treatment. That said, the validation procedures for the MSDA biomarkers (and particularly for the ones that were significant in this analysis such as GFAP) demonstrated excellent recovery and no interference with the most commonly used DMTs, common medications, endogenous interferents and heterophilic antibodies.^19^ Detailed results for each biomarker of the assay regarding their interference and recovery can be seen elsewhere.^19^ More frequent and serial blood draws can better establish the chronological timeline regarding the relationship between proteomic data with MRI-based pathological changes. Another potential limitation is the measurement of full-length proteins with potentially unknown long-term stability. Alterative assays that quantify soluble cleavage products have significantly greater stability. That said, in the studies used to develop and validate the custom assay panel, statistically meaningful associations of biomarkers with multiple multiple sclerosis end-points were observed using samples that had been stored at −80°C for extended periods of time (up to 5 and more years of follow-up).^19^ This suggests that the target epitopes for the proteins that were selected for inclusion in the custom assay panel and the final algorithm were sufficiently stable to derive clinically meaningful insights. Serial clinical tests could also better characterize the study cohort and allow the calculation of better-suited disability measures. Recent development of newer composite outcome measures such as progression independent of relapse activity (PIRA) and relapse-associated worsening has demonstrated that people with multiple sclerosis can progress through different underlying mechanisms.^55^ Along these lines, we emulated a similar proxy comparison in our study sample and showcased that people with multiple sclerosis that progressed without the presence of any relapse had significantly greater baseline NfL levels. The presence of PIRA in many relapsing multiple sclerosis cases also challenges the concept and distinction of disease subtypes. Composite clinical measures that better classify the disease could significantly improve the association between phenotypical characterization and proteomic measures in multiple sclerosis. Similarly, unified use of a single DP definition represents a limitation when utilized in more heterogeneous multiple sclerosis groups. Whereas clinically small DP changes in the relapsing group (for example EDSS change from 2.0 to 3.0) could occur more commonly and at shorter intervals, the DP in people with multiple sclerosis occurs at longer periods and has a significant ‘immortal time’ bias.^56^ All these factors lead to comparably higher DP rates in relapsing multiple sclerosis and lower DP rates in the people with progressive multiple sclerosis. The rate of DP in our people with multiple sclerosis mirrors the one seen in other studies of similar follow-up. For example, the initial IFN-β arm of the OPERA trials experienced more than 20% DP over the 5-year open-label extension.^57^ A similar rate of DP was seen in the CIS-based IFN-β trial (BENEFIT), where ∼25% experienced disability worsening over 5-year follow-up.^58^ Newer, more effective treatments and better risk classifications have recently resulted in a lower rate of progression in the relapsing forms of multiple sclerosis (only 11.4% of ozanimod-treated people with multiple sclerosis experienced DP over 5-year follow-up; DAYBREAK trial).^59,60^ Lastly, all our findings are based on group comparisons and do not reflect individual proteomic changes. The individual predictive ability of the assay can be addressed by creating large multiple sclerosis-based proteomic reference data, which will allow age, sex and disease duration-matched comparisons.

In conclusion, multiple serum biomarkers such as GFAP, MOG and NfL levels were consistently associated with future widespread microstructural axonal changes as measured by DTI-based MRI. Baseline serum GFAP levels can predict future DP. Assays providing information for multiple biomarkers at once can provide better risk stratification of future multiple sclerosis disability and identify people with multiple sclerosis with greater future myelin/axonal destruction. Differential biomarkers may be better indicators of volumetric versus microstructural pathology in multiple sclerosis. Creating and validating a neurodegenerative composite biomarker score consisting of the most predictive measures could further improve the utility of such multivariate assays.

## Supplementary Material

fcad183_Supplementary_Data

## Data Availability

The data that support the findings of this study are available upon reasonable request from the corresponding author.

## Abbreviations

APLP1 =: amyloid beta precursor like protein 1
BBB =: blood–brain barrier
BMI =: body mass index
CCL20 =: MIP-3 alpha
CD6 =: Cluster of Differentiation 6
CDCP1 =: CUB domain-containing protein 1
CIS =: clinically isolated syndrome
CNTN2 =: contactin 2
COL4A1 =: collagen alpha-1 (IV) chain
CV =: coefficients of variability
CXCL13 =: C-X-C Motif Chemokine Ligand 13, BLC
CXCL9 =: monokine induced by gamma interferon MIG
DMT =: disease-modifying therapy
DP =: disability progression
DTI =: diffusion tensor imaging
EDSS =: Expanded Disability Status Scale
EMA =: European Medicines Agency
EPI =: echo-planar imaging
FA =: fractional anisotropy
FDR =: false discovery rate
FDT =: FMRIB’s Diffusion ToolBox
FLAIR =: fluid attenuated inverse recovery
FLRT2 =: leucine-rich repeat transmembrane protein
Gd =: gadolinium
GFAP =: glial fibrillary acidic protein
GH =: Somatotropin, growth hormone
GM =: grey matter
IL-12B =: interleukin 12B
IRB =: Institutional Review Board
JIM =: Java Image Manipulation
LV =: lesion volume
MD =: mean diffusivity
MOG =: myelin-oligodendrocyte glycoprotein
MSDA =: multiple sclerosis disease activity
NABT =: normal-appearing brain tissue
NAWM =: normal-appearing white matter
NfL =: neurofilament light chain
NMOSD =: neuromyelitis optica spectrum disorder
OPG =: osteoprotegerin, TNFRSF11B
OPN =: osteopontin
PBVC =: per cent brain volume change
PEA =: Proximity Extension Assay
PIRA =: progression independent of relapse activity
PRTG =: protogenin
RAW =: relapse-associated worsening
ROI =: region of interest
SERPINA9 =: serpin family A member 9
SIENA =: Structural Image Evaluation, using Normalisation, of Atrophy
TNFRSF10A =: TRAILR1, DR5—Death Receptor 5
TNFSF13B / BAFF =: B-cell activating factor
VCAN =: versican, versican proteoglycan
WB =: whole brain
