# Supplementary material for: Proteomics and relationship with axonal pathology in multiple sclerosis: 5-year diffusion tensor imaging study
Source: Brain Commun. 2023 Jun 13;5(3):fcad183. doi: 10.1093/braincomms/fcad183 (PMC10288551; doi:10.1093/braincomms/fcad183)
Supplement: fcad183_Supplementary_Data [file fcad183_supplementary_data.pdf]

Supplemental Figure 1. Longitudinal correlation matrix (heatmap) between baseline proteomics and diffusion tensor imaging outcomes in CIS/RRMS and PMS.

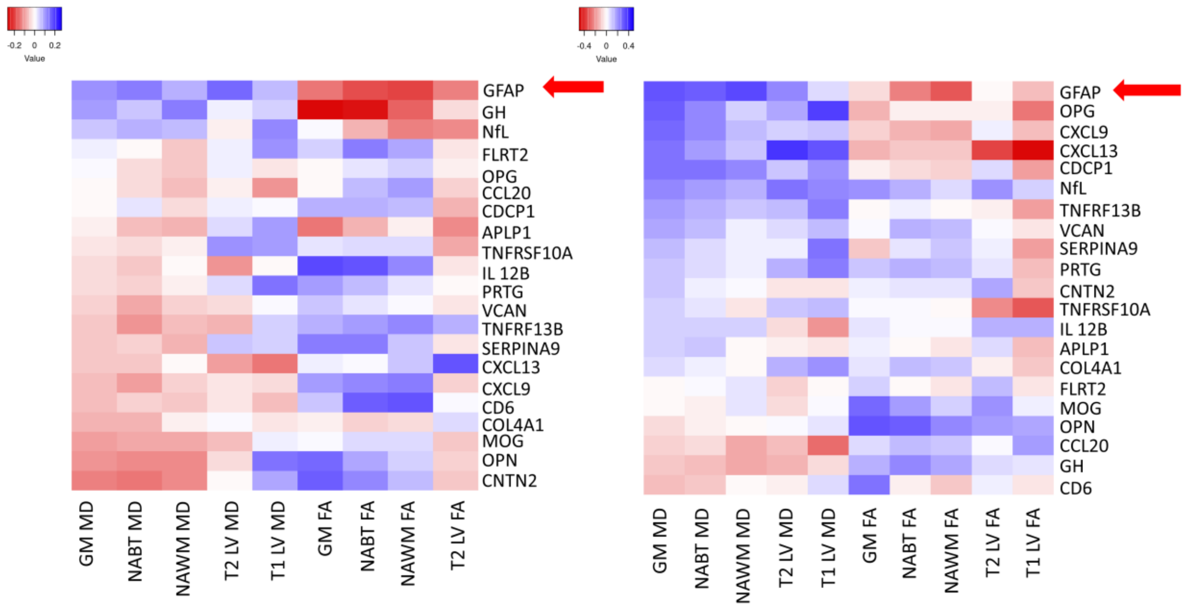

Legend: Correlation data (Spearman's ranked correlation coefficients) was performed in 132 pwCIS/RRMS and 49 pwPMS. The magnitude of the correlation coefficient is shown in the legend. Red squared demonstrate positive correlation coefficients and blue squares demonstrate negative correlation coefficients. Red arrows indicate biomarkers with significant correlations with DTI-based measures.

CIS/RRMS – clinically isolated syndrome/relapsing-remitting multiple sclerosis, PMS – progressive multiple sclerosis, MD – mean diffusivity, FA – fractional anisotropy, GM – gray matter, NABT – normal-appearing brain tissue, NAWM – normal-appearing white matter, LV – lesion volume.RRMS – relapsing-remitting multiple sclerosis, PMS – progressive multiple sclerosis, APLP1 – amyloid beta precursor like protein 1, CCL20 – C-C motif chemokine ligand 20, CD6 – T-cell differentiation antigen CD6, CDCP1 – CUB domain containing protein 1, CNTN2 – contactin 2, COL4A1 – collagen type IV alpha 1, CXCL13 – chemokine ligand 13, CXCL9 – chemokine ligand 9, FLRT2 – fibronectin leucine rich transmembrane protein 2, GFAP – glial fibrillary acidic protein, GH – growth hormone, IL-12B – interleukin 12 subunit B, MOG – myelin oligodendrocyte glycoprotein, NfL – neurofilament light chain, OPG – osteoprotegerin, OPN – osteopontin, PRTG – protogenin precursor, SERPINA9 – serpin family A member A, TNFRSF10A – tumor necrotic factor receptor superfamily member 10a, TNFSF13b – tumor necrotic factor superfamily member 13b, VCAN – versican.

Supplemental Table 1. Proteomics data in relapsing-remitting and progressive multiple sclerosis at baseline and follow-up timepoints.

Proteomic measure (median and IQR)	At baseline (n=202)		p-value	At follow-up (n=143)		p-value
	CIS/RRMS	PMS		CIS/RRMS	PMS	
Age at baseline	44.1 (10.6)	55.3 (7.9)	**<0.001**	43.3 (10.5)	54.6 (8.1)	**<0.001**
APLP1 in ng/mL	11.1 (8.7-13.9)	12.1 (9.5-13.4)	0.283	12.5 (10.6-14.5)	12.9 (10.3-14.6)	0.717
CCL20	10 (5.8-18.6)	8.8 (5.8-16.1)	0.628	14.2 (6.5-38.1)	20.1 (10.2-37.5)	0.166
CD6	132.1 (100-172.9)	130.4 (90-187.8)	0.532	140.5 (107.4-183.6)	146.3 (114.7-180.9)	0.544
CDCP1	102.4 (69.3-142.2)	114 (80-141.5)	0.226	93.5 (75.1-139.8)	139.7 (106.1-171.5)	**0.002**
CNTN2 in ng/mL	1.6 (1.2-2.2)	1.9 (1.4-2.4)	0.058	1.9 (1.4-2.7)	2.1 (1.8-2.9)	0.064
COL4A1 in ng/mL	1.3 (0.9-2.1)	12.1 (1.0-1.9)	0.553	1.1 (0.9-1.6)	1.3 (0.9-1.9)	0.125
CXCL13	49.1 (41-67.3)	50.3 (36.4-65.1)	0.644	50.7 (40.7-65)	56 (42.8-81)	0.223
CXCL9	48.2 (33.7-84)	70.9 (49.9-89.7)	**0.009**	53.5 (34.9-97.5)	79.4 (48.3-111.4)	**0.008**
FLRT2	108 (87.9-134.4)	113.8 (96.1-129)	0.507	115.8 (97.1-132.5)	121.4 (100.5-136.8)	0.395
GFAP	105.8 (77.4-147)	145.8 (115-177.3)	**<0.001**	116.1 (88.7-149.1)	163.8 (120.1-203.6)	**<0.001**
GH	175.8 (73.1-460.7)	185.3 (69-1134.2)	0.368	154.6 (63.4-498.5)	183.7 (69.5-516.4)	0.544
IL-12B	108.8 (78.5-179.6)	124.4 (74.6-172)	0.883	107.5 (76.9-150.3)	121.6 (79.7-209.7)	0.306
MOG	27.1 (21.4-33.3)	28.4 (22.6-33.7)	0.456	30.8 (24.1-37.4)	32.2 (23.4-38.2)	0.919
NfL	8.6 (6.4-12.3)	12.5 (9.5-15.5)	**<0.001**	10.4 (7.6-14.4)	13.7 (10.7-20.5)	**<0.001**
OPG	742.9 (589.2-923)	827.4 (613.7-940)	0.348	811.9 (677-1043.3)	842.8 (646.9-1089.8)	0.833
OPN in ng/mL	18.8 (15.1-25.1)	19.7 (15.1-23.7)	0.817	21.2 (15.9-26.9)	21.7 (18.5-30.0)	0.378
PRTG	127.9 (108.1-148.2)	127.3 (114.4-148.5)	0.87	133.8 (113.1-154.6)	133.4 (111.2-153)	0.846
SERPINA9	50 (34.6-75.9)	57.1 (37.6-90.6)	0.111	53.5 (33.6-72.5)	61.2 (37-99.5)	0.116
TNFRSF10A	5.6 (4.7-7.3)	6 (4.9-7.1)	0.617	6 (5-7.4)	6.8 (5.5-8.9)	**0.044**
TNFSF13B in ng/mL	4.8 (4.1-5.9)	4.6 (3.9-5.5)	0.135	5.1 (4.1-6.1)	5.0 (4.4-6.3)	0.739
VCAN	436.1 (367.1-512.5)	451 (387.6-537)	0.404	460.8 (358.7-527.8)	491.3 (421.9-532.7)	0.039

Legend: CIS – clinically isolated syndrome, RRMS – relapsing-remitting multiple sclerosis, PMS – progressive multiple sclerosis, IQR – interquartile range, APLP1 – amyloid beta precursor like protein 1, CCL20 – C-C motif chemokine ligand 20, CD6 – T-cell differentiation antigen CD6, CDCP1 – CUB domain containing protein 1, CNTN2 – contactin 2, COL4A1 – collagen type IV alpha 1, CXCL13 – chemokine ligand 13, CXCL9 – chemokine ligand 9, FLRT2 – fibronectin leucine rich transmembrane protein 2, GFAP – glial fibrillary acidic protein, GH – growth hormone, IL-12B – interleukin 12 subunit B, MOG – myelin oligodendrocyte glycoprotein, NfL – neurofilament light chain, OPG – osteoprotegerin, OPN – osteopontin, PRTG – protogenin precursor, SERPINA9 – serpin family A member A, TNFRSF10A – tumor necrotic factor receptor superfamily member 10a, TNFSF13b – tumor necrotic factor superfamily member 13b, VCAN - versican

All measures are shown in picogram in milliliter (pg/mL) unless specified in the Table. All measures are shown as median and interquartile range (IQR). All comparisons were performed using paired non-parametric Mann-Whitney U test. P-values lower than 0.05 were considered statistically significant and shown in bold. The MS subtype classification is based on the specific timepoint.

Supplemental Table 2. Differences in proteomic data based on the baseline disease modifying therapy in people with multiple sclerosis.

Baseline proteomic data	No DMT (n=46)	IFN-β (n=85)	Glatiramer acetate (n=37)	Natalizumab (n=29)	Off-label DMT (n=5)	p-value
Age at baseline	48.9 (11.0)	46.9 (12.0)	47.9 (10.4)	43.1 (8.9)	50.4 (9.8)	0.223
APLP1 in ng/mL	11.4 (9.4-13.9)	11.1 (8.5-14.)	11.8 (9.7-13.7)	11.2 (8.8-12.9)	13.2 (9.8-16.7)	0.771
CCL20	9 (5.4-22.9)	11.4 (6.5-19.4)	9.7 (6-16.2)	6 (3.9-12.1)	9.2 (6.8-15.3)	0.129
CD6	142.4 (122-195.3)	121.8 (88.7-168.7)	152.2 (112-201.9)	117.3 (93.9-167.6)	105.5 (75.5-135.8)	**0.018**
CDCP1	112.2 (73.9-137.5)	108.1 (82.5-163.6)	102.2 (66.9-131.5)	81.9 (55.1-130.7)	102.7 (64.6-129.9)	**0.025**
CNTN2 in ng/mL	1.8 (1.2-2.2)	1.8 (1.3-2.4)	1.9 (1.4-2.6)	1.4 (1.1-1.8)	1.5 (1.3-2.4)	0.203
COL4A1 in ng/mL	1.2 (0.9-2.1)	1.4 (1.1-1.9)	1.0 (0.9-1.9)	1.3 (0.9-2.1)	1.3 (0.9-7.6)	0.266
CXCL13	50.2 (40.5-73.3)	53.3 (42.6-69.8)	50.5 (42.4-67.6)	35.9 (29.3-44.5)	60.3 (49-104)	**<0.001**
CXCL9	50.8 (38-87.8)	52.8 (36.2-82.2)	94.5 (58.5-172.2)	33 (25.1-43.8)	53.7 (37-63.4)	**<0.001**
FLRT2	118.5 (99.2-134.4)	103.3 (82.5-127.1)	115.8 (93.7-129)	107.6 (87.9-131.9)	137.8 (107.2-146.6)	0.336
GFAP	114.9 (93.6-150.2)	115.8 (77.6-165)	122.8 (88-165.9)	104.4 (82.2-132.2)	183 (158.6-223.7)	0.073
GH	222.1 (71.6-750.9)	171.8 (62.2-1011.7)	141.4 (64.3-334.6)	257.3 (119-847)	89.1 (55.6-2963.7)	0.469
IL-12B	102.7 (72.8-138.5)	116.1 (81.4-188.2)	119.3 (73.4-193.7)	111.1 (88.2-168.9)	75.4 (70.3-148.7)	0.378
MOG	28.9 (24.5-34.8)	27.1 (20.7-32.9)	29.6 (23-35)	23.7 (19.6-29.3)	30.8 (25-39.9)	0.115
NfL	10.1 (7.7-12.7)	10 (6.7-14.6)	10.2 (7.5-13.3)	7.7 (5.6-11.6)	13.1 (12.6-53.2)	**0.022**
OPG	765.6 (590-887.2)	796.6 (578.9-1013.1)	746.1 (588-931)	659.2 (582.6-868.9)	877 (707.2-1008.5)	0.434
OPN in ng/mL	20.0 (15.6-23.4)	18.9 (14.8-25.4)	20.6 (15.2-25.4)	18.2 (143-27.5)	23.6 (17.1-33.8)	0.598
PRTG	127 (107.9-148)	129.1 (108.4-148.3)	126.6 (109.4-148.9)	121.5 (105.1-147.3)	139.4 (116.7-180.5)	0.672
SERPINA9	52.7 (36.3-91.5)	54.7 (35.6-78.6)	51.9 (37.3-83.9)	37.6 (32.8-61.3)	35.3 (22.6-69.3)	0.119
TNFRSF10A	6.1 (4.8-7.3)	5.9 (4.9-8.1)	5.8 (5.1-7.2)	4.5 (4.2-5.3)	5 (4.4-7.4)	**0.005**
TNFSF13B in ng/mL	4.6 (4.1-5.4)	5.3 (4.3-6.6)	4.8 (4.0-5.9)	4.2 (3.7-5.0)	4.9 (4.6-6.3)	**0.04**
VCAN	421.8 (366.4-473.7)	443.7 (367.6-520.3)	464.7 (392-512.7)	415.5 (364.5-494.3)	594.9 (557.8-637.3)	**0.036**
APLP1 % change	2.6 (-16.2-30.1)	0.3 (-15.7-19.6)	10.6 (-7.5-28)	2.6 (-25.4-24.4)	-	0.56
CCL20 % change	89.2 (-20.2-207)	64.7 (-48.5-241.9)	50.6 (-28.7-191.1)	89.3 (-22.8-309.3)	-	0.876
CD6 % change	7.7 (-16-21.6)	-7.7 (-28-38.6)	4.9 (-24.6-46.8)	17 (-36.6-38.2)	-	0.947
CDCP1 % change	16.9 (-18.3-46.8)	9.8 (-18.2-31.8)	7.5 (-16.2-49.6)	27.4 (-24.4-82.2)	-	0.805
CNTN2 % change	9.3 (-12.6-36.8)	5.9 (-19.6-28.4)	10.1 (-12.7-34.1)	7.5 (-9.3-23.4)	-	0.919
COL4A1 % change	3.6 (-37.6-55.7)	-4 (-31.1-13.1)	2.7 (-31.5-61.8)	-5.2 (-46.1-38.3)	-	0.644
CXCL13 % change	2.6 (-20.7-41.6)	2.7 (-34.8-32)	2.2 (-19.6-24.2)	29.9 (-17.5-57.5)	-	0.361
CXCL9 % change	-0.9 (-23.8-35.7)	5 (-23.8-43.8)	-2 (-36.2-44.4)	45.6 (12.7-192.2)	-	0.043
FLRT2 % change	-0.2 (-15.1-14.9)	4.1 (-21.7-19.8)	2.5 (-8.5-15.4)	7.9 (-18.8-27.8)	-	0.985
GFAP % change	13 (-8.7-39.5)	4.7 (-20.5-41.6)	13.7 (-10.5-34.3)	-1.8 (-12.9-33.7)	-	0.787
GH % change	26.7 (-53.1-200.8)	-39.5 (-75.9-166)	32.7 (-64.4-166.4)	-47.7 (-82.4-23.5)	-	0.179
IL-12B % change	-4.7 (-25.6-22.4)	-1.8 (-22.1-28.5)	-0.3 (-21.4-19.8)	4.8 (-32.3-31.5)	-	0.95
MOG % change	5.6 (-14.9-33)	7.6 (-21-41.2)	14.8 (-4.9-39.5)	-6.3 (-15.5-32.6)	-	0.619
NfL % change	10.2 (-10.7-37.6)	12.8 (-21.3-43.6)	16 (-6.2-49.4)	7.3 (-19.1-98.3)	-	0.823
OPG % change	5.7 (-11.5-29.1)	9.7 (-22.9-28.8)	3.2 (-12.7-27.3)	-0.9 (-14.6-15.6)	-	0.631
OPN % change	1.9 (-22.9-53.8)	7.8 (-19.9-27.2)	15.8 (-19.9-54.9)	21.7 (-3.4-51)	-	0.878
PRTG % change	1.1 (-8.2-14.3)	0.8 (-11.9-13.1)	2.9 (-17-12.4)	-1.7 (-10.8-25.4)	-	0.896
SERPINA9 % change	9.8 (-30.8-33.2)	-1.7 (-31.9-45)	-16.6 (-41.8-21.3)	2.6 (-41.2-70.8)	-	0.637
TNFRSF10A % change	1.7 (-15-29.3)	1.4 (-20.9-23.4)	6.2 (-7.8-31.2)	18 (-16.3-39.6)	-	0.489
TNFSF13B % change	0.9 (-15.4-25.8)	0.2 (-18.4-20.3)	-1.2 (-21.6-14.9)	7 (-11-11.9)	-	0.726
VCAN % change	6.1 (-12.9-20.9)	-3.5 (-17.3-13.6)	5.7 (-9.2-9.3)	-4 (-20.6-32.3)	-	0.672

Legend: DMT – disease modifying therapy, IFN-β – interferon-beta, APLP1 – amyloid beta precursor like protein 1, CCL20 – C-C motif chemokine ligand 20, CD6 – T-cell differentiation antigen CD6, CDCP1 – CUB domain containing protein 1, CNTN2 – contactin 2, COL4A1 – collagen type IV alpha 1, CXCL13 – chemokine ligand 13, CXCL9 – chemokine ligand 9, FLRT2 – fibronectin leucine rich transmembrane protein 2, GFAP – glial fibrillary acidic protein, GH – growth hormone, IL-12B – interleukin 12 subunit B, MOG – myelin oligodendrocyte glycoprotein, NfL – neurofilament light chain, OPG – osteoprotegerin, OPN – osteopontin, PRTG – protogenin precursor, SERPINA9 – serpin family A member A, TNFRSF10A – tumor necrotic factor receptor superfamily member 10a, TNFSF13b – tumor necrotic factor superfamily member 13b, VCAN – versican

Age is shown in years and as mean (standard deviation; SD). All proteomic data are shown as median % change (interquartile range; IQR). Age is compared using one-way analysis of variance (ANOVA), whereas proteomic data was compared using Kruskal-Wallis H test. P-value lower than 0.05 was considered statistically significant and shown in bold.

Supplemental Table 3. Percent change in proteomic data based on the change in disease modifying therapy in 143 people with multiple sclerosis with available longitudinal sample at follow-up.

Proteomic data	Remained on the same DMT (n=92)	Started on DMT (n=13)	Switched DMT (n=31)	Stopped DMT (n=7)	Age-adjusted ANCOVA p-value
Age at baseline	48.5 (10.5)	45.9 (11.1)	43.3 (11.2)	50.5 (13.6)	**0.028**
APLP1	2.2 (-15.7-27.3)	13.7 (-2.4-37.4)	1.6 (-18.2-15.4)	-10.8 (-21.2-19.6)	0.416
CCL20	35.7 (-37.1-187.5)	131.3 (37.5-347.2)	100.1 (-23.4-262.3)	169.9 (-57.9-353.7)	0.628
CD6	8.7 (-22.3-43)	10.3 (-33.7-23.1)	-17.9 (-35.6-27.6)	7.7 (-14.6-41.9)	0.437
CDCP1	11.7 (-17.6-43.3)	32 (-12.5-64.6)	7.3 (-24.4-53.1)	9 (-15.6-32.4)	0.604
CNTN2	9.3 (-12.6-32.2)	38.4 (-5-68.6)	0.4 (-15.5-19.8)	9.6 (-19.4-26.1)	0.225
COL4A1	-7.9 (-36.6-16.1)	30.7 (15.4-94.9)	-0.5 (-25.8-33.2)	-22.8 (-58.1–2.4)	0.25
CXCL13	1 (-21.9-24.9)	36.2 (-8.7-69.4)	6.8 (-35.1-47.2)	12.4 (-29.4-32.1)	0.15
CXCL9	4.9 (-32.8-52.2)	5 (-17-43.9)	17.9 (-9-89.4)	12 (-4-71.7)	0.404
FLRT2	0.2 (-14.8-14.9)	14.1 (-8.8-37)	8.1 (-18.8-27.8)	-7.3 (-9.4-19.1)	0.459
GFAP	12.6 (-17.3-41.6)	18.1 (5.2-54.9)	-1.8 (-14.2-15.5)	2.6 (-1.6-26.8)	0.339
GH	-16.6 (-74.2-150.2)	28.6 (-51.3-1081)	-12.7 (-71.5-166.4)	-80.9 (-96.5–55.1)	0.408
IL-12B	0.3 (-23.4-27.5)	-10.8 (-27.2-32.5)	-12.2 (-32.3-21.8)	3.9 (-19.9-76.5)	0.592
MOG	11.6 (-18.6-36.6)	0.1 (-7-50.7)	-0.6 (-15.5-32.6)	12.4 (-21.1-16.1)	0.379
NfL	7.8 (-19.5-39.7)	8.5 (-18-38.3)	21.8 (-7.8-51.3)	20.7 (-7.8-104.6)	0.823
OPG	4.3 (-15.3-27.1)	20.7 (-8.6-48.2)	0.9 (-18.2-25.9)	15.9 (-28.3-28.8)	0.692
OPN	6.3 (-20.2-47.5)	25 (-19.7-98.1)	20.1 (-6.8-27.6)	-19.6 (-34.8-14.2)	0.397
PRTG	0.2 (-11.9-13)	7 (-7.1-20.4)	-1.8 (-13-19.1)	-0.5 (-20.2-9.2)	0.639
SERPINA9	-4.1 (-34.6-34.6)	9.8 (-51.5-28.3)	-12.4 (-33.8-50.3)	16 (-4.2-103.8)	0.857
TNFRSF10A	3.8 (-12.1-28.7)	18.7 (-8.3-43.1)	-6.3 (-12.7-29.1)	3.5 (-21-8.1)	0.404
TNFSF13B	-0.3 (-17.2-18.4)	30.9 (-10.3-56)	3.4 (-14.7-11.9)	-0.8 (-28.9-12.3)	0.06
VCAN	0.2 (-12.4-14.7)	20.9 (1.9-37.4)	-8.1 (-19.6-12)	-3 (-28.3-14.8)	0.178

Legend: DMT – disease modifying therapy, APLP1 – amyloid beta precursor like protein 1, CCL20 – C-C motif chemokine ligand 20, CD6 – T-cell differentiation antigen CD6, CDCP1 – CUB domain containing protein 1, CNTN2 – contactin 2, COL4A1 – collagen type IV alpha 1, CXCL13 – chemokine ligand 13, CXCL9 – chemokine ligand 9, FLRT2 – fibronectin leucine rich transmembrane protein 2, GFAP – glial fibrillary acidic protein, GH – growth hormone, IL-12B – interleukin 12 subunit B, MOG – myelin oligodendrocyte glycoprotein, NfL – neurofilament light chain, OPG – osteoprotegerin, OPN – osteopontin, PRTG – protogenin precursor, SERPINA9 – serpin family A member A, TNFRSF10A – tumor necrotic factor receptor superfamily member 10a, TNFSF13b – tumor necrotic factor superfamily member 13b, VCAN – versican

Age is shown in years and as mean (standard deviation; SD). All proteomic data are shown as median % change (interquartile range; IQR). P-value lower than 0.05 was considered statistically significant and shown in bold.

Supplemental Table 4. Linear step-wise regression determining associations between baseline proteomics and conventional MRI measures in people with multiple sclerosis.

Conventional MRI measures	Predictors	R^2	Standardized β	p-value
Absolute T2-LV change	IL-12B	0.089	0299	**<0.001**
PBVC	GFAP	0.072	-0.296	**0.001**
	PRTG	0.123	0.207	**0.009**
	NfL	0.149	-0.266	**0.003**
	MOG	0.176	0.260	**0.010**
GM atrophy	NfL	0.040	-0.197	**0.016**
	PRTG	0.086	0.273	**0.003**
	OPN	0.146	-0.329	**<0.001**

Legend: LV – lesion volume, PBVC – percent brain volume change, GM – gray matter. GFAP – glial fibrillary acidic protein, OPN – osteopontin, NfL – neurofilament light chain, MOG – myelin oligodendrocyte glycoprotein PRTG – protogenin precursor, IL-12B – interleukin 12 subunit B.

Step-wise regression model was utilized where conventional MRI measures were considered as a dependent variable and all baseline proteomics data were added as independent predictors. All proteomic data was logarithmically transformed Log(10). In addition to the proteomics data, the age, sex and BMI of the pwMS was also included. P-values lower than 0.05 were considered as statistically significant and shown in bold.

Supplemental Table 5. Linear step-wise regression determining cross-sectional associations between follow-up proteomics and microstructural DTI-based measures in people with multiple sclerosis at the follow-up visit in 143 available serum samples at follow-up.

Fractional anisotropy (FA)	Predictors	R^2	Standardized β	p-value
NABT FA	BMI	0.08	0.264	**0.002**
	Sex	0.128	0.248	**0.003**
	NfL	0.164	-0.322	**0.002**
	MOG	0.196	0.224	**0.027**
NAWM FA	GFAP	0.092	-0.314	**<0.001**
GM FA	Sex	0.053	0.242	**0.005**
	Age	0.103	0.225	**0.009**
T2-LV FA	Age	0.087	-0.184	**0.044**
	IL-12B	0.117	-0.205	**0.018**
	COL4A1	0.149	0.244	**0.007**
	GFAP	0.177	-0.191	**0.044**
T1-LV FA	Sex	0.091	0.304	**0.002**
	CNTN2	0.13	-0.336	**0.004**
	PRTG	0.177	0.257	**0.027**

Mean diffusivity (MD)	Predictors	R^2	Standardized β	p-value
NABT MD	CXCL13	0.059	0.188	**0.036**
	GFAP	0.091	0.188	**0.036**
NAWM MD	NfL	0.058	0.241	**0.006**
GM MD	GFAP	0.063	0.251	**0.005**
T2-LV MD	GFAP	0.073	0.362	**0.001**
	MOG	0.104	-0.282	**0.008**
	TNFRSF10a	0.144	0.219	**0.018**
T1-LV MD	NfL	0.053	0.415	**0.002**
	VCAN	0.106	-0.295	**0.023**

Legend: BMI – body mass index, GFAP - glial fibrillary acidic protein, OPN - osteopontin, CXCL13 – chemokine ligand 13, CNTN2 - contactin 2, NfL – neurofilament light chain, MOG - myelin oligodendrocyte glycoprotein, TNFRSF10a - tumor necrotic factor receptor superfamily member 10a, VCAN – versican, DTI – diffusion tensor imaging, MD – mean diffusivity, FA – fractional anisotropy, GM – gray matter, NABT – normal-appearing brain tissue, NAWM – normal-appearing white matter, LV – lesion volume.

Step-wise regression model was utilized where the DTI-based measures were considered as a dependent variable and all proteomics data were added as independent predictors. All proteomic data was logarithmically transformed Log(10). In addition to the proteomics data, the age, sex and BMI of the pwMS was also included. P-values lower than 0.05 were considered as statistically significant and shown in bold.
